# Promotion of knowledge transfer and retention in year 2 medical students using an online training exercise

**DOI:** 10.1007/s10459-021-10037-y

**Published:** 2021-03-09

**Authors:** Lucy V. Rosby, Henk G. Schmidt, Gerald J. S. Tan, Naomi Low-Beer, Silvia Mamede, Laura Zwaan, Jerome I. Rotgans

**Affiliations:** 1 Lee Kong Chian School of Medicine, Nanyang Technological University, Singapore, Singapore; 2 Erasmus University Rotterdam, Rotterdam, The Netherlands; 3 Erasmus Medical Center, Rotterdam, The Netherlands

**Keywords:** Dual-process theory, System-1 processing, Transfer of learning, Knowledge transfer, Knowledge retention, Diagnostic reasoning, Medical student, Radiology

## Abstract

It was recently shown that novice medical students could be trained to demonstrate the speed-to-diagnosis and diagnostic accuracy typical of System-1-type reasoning. However, the effectiveness of this training can only be fully evaluated when considering the extent to which knowledge transfer and long-term retention occur as a result, the former of which is known to be notoriously difficult to achieve. This study aimed to investigate whether knowledge learned during an online training exercise for chest X-ray diagnosis promoted either knowledge transfer or retention, or both. Second year medical students were presented with, and trained to recognise the features of four chest X-ray conditions. Subsequently, they were shown the four trained-for cases again as well as different representations of the same conditions varying in the number of common elements and asked to provide a diagnosis, to test for near-transfer (four cases) and far-transfer (four cases) of knowledge. They were also shown four completely new conditions to diagnose. Two weeks later they were asked to diagnose the 16 aforementioned cases again to assess for knowledge retention. Dependent variables were diagnostic accuracy and time-to-diagnosis. Thirty-six students volunteered. Trained-for cases were diagnosed most accurately and with most speed (mean score = 3.75/4, mean time = 4.95 s). When assessing knowledge transfer, participants were able to diagnose near-transfer cases more accurately (mean score = 2.08/4, mean time = 15.77 s) than far-transfer cases (mean score = 1.31/4, mean time = 18.80 s), which showed similar results to those conditions previously unseen (mean score = 0.72/4, mean time = 19.46 s). Retention tests showed a similar pattern but accuracy scores were lower overall. This study demonstrates that it is possible to successfully promote knowledge transfer and retention in Year 2 medical students, using an online training exercise involving diagnosis of chest X-rays, and is one of the few studies to provide evidence of actual knowledge transfer.

## Introduction

Accurate decision-making lies at the heart of successful medical practice, as this ultimately leads to the correct diagnosis and management of a patient’s condition. The way doctors make these diagnostic decisions is explained largely by clinical reasoning, which has become the focus of much research in recent years in response to the finding that diagnostic errors explain over 8% of adverse events in medicine and up to 30% of malpractice claims (Nendaz & Perrier, 2012). It has been estimated that up to 75% of diagnostic mistakes are a result of thinking errors, attributed to in part insufficient knowledge and an inability to fully understand data gathered (Thammasitboon & Cutrer, 2013). The dominant theory which explains the way doctors make their clinical decisions is Dual-Process Theory, referring to two distinct thinking processes: System 1, which is fast, autonomous (Evans & Stanovich, 2013) and often associated with expertise (Evans & Stanovich, 2013; Norman et al., 2014) allowing doctors to make decisions based on stored ‘illness scripts’ and pattern recognition (Charlin et al., 2007; Schmidt & Rikers, 2007) and System 2, the slow, analytical, higher order process used to address complex problems or when expertise is lacking (Evans & Stanovich, 2013).

Given the importance of diagnostic reasoning skills in medicine, it would be natural to assume that such skills are specifically taught and trained for during medical school. However, this is the case only to a limited extent. More often, it is expected that medical students will pick these skills up during the course of their training (Schmidt & Mamede, 2015). In part, the reason this is often lacking in formal medical education curricula is due to uncertainty about how to effectively teach it (Eva, 2005). When it is taught, the focus is often on the analytical component of decision making—System-2 thinking—quite possibly because medical students are considered novices who may need to take this approach, as opposed to the heuristic, non-analytical System-1, more often utilised by experts with more knowledge and experience (Norman et al., 2007).

In a recent study by Rosby et al. (2018), it was shown that novice medical students were able to develop System-1-type thinking as often employed by experts (Norman et al., 2014), albeit in a limited domain, and in response to a short online training exercise diagnosing chest x-rays. In this study, they were shown a series of 8 online cases consisting of chest x-rays without clinical vignettes. Participants were shown half of the cases repeatedly during a training phase and the other half only twice, once in an initial familiarisation phase and then again in a final test phase. During the test phase, they were shown all 8 cases, being asked to provide a diagnosis as a free text answer. Dependent variables were diagnostic accuracy and response time. Diagnostic accuracy and time-to-diagnosis for the trained cases showed the typical pattern found in experts: high diagnostic accuracy and short decision times, relative to the untrained cases. However, these researchers failed to demonstrate whether this training allowed participants to use the knowledge they had gained from the exercise in cases *beyond* those specifically trained for. In other words, did this exercise simply train the participants to pattern-recognise the specific images seen during their training, *or* had they identified the key features of the conditions trained for and were able to *transfer* this knowledge to diagnose different x-rays representing the same condition? This transfer of knowledge is key in determining whether exercises such as the one described can be used to effectively train for non-analytical reasoning processes. This is is what we aimed to explore in the study presented here.

Although the first studies of transfer of learning were already conducted more than 100 years ago (Thorndike & Woodworth, 1901), the nature of transfer, and the conditions under which it occurs, still is a controversial and largely unresolved problem within psychology, despite recent attempts to resolve the issue (e.g., Cormier & Hagman, 2014; Haskell, 2001) A major problem is that successful knowledge transfer rarely occurs (Gick & Holyoak, 1980; Haskell, 2001). This is commonly referred to as the “inert knowledge” problem (Whitehead, 1929); people have the knowledge but are unable to apply it in new situations. This failure to transfer is particularly problematic in the workplace, where employers often complain that new hires cannot carry out tasks that they have allegedly been trained for (Larsen-Freeman, 2013). This is in particular pressing in the context of medicine where the ability to transfer what one has learned and apply it to other clinical problems is paramount to successful diagnosis and safe management of patients. A second problem is one of definition. Here points of view differ widely. Perkins and Salomon (1992), for instance, speak of reflexive or low road transfer that involves the triggering of well-practiced routines by stimulus conditions similar to those in the learning context. Mindful or high road transfer involves deliberate effortful abstraction and a search for connections. They also popularized the concepts of *near* and *far transfer*, but are ambiguous about whether this difference is similar to their low-road-high-road distinction. Gagne (1970) distinguishes between vertical and lateral transfer. Vertical transfer occurs when a skill or knowledge contributes directly to the subsequent acquisition or performance of a superordinate task or skill. Lateral involves “a kind of generalization that spreads over a broad set of situations at roughly the same level of complexity” (p. 231). In the context of medical education, Kulasegaram et al. (2017) distinguishes between near transfer (applying basic science knowledge to explain pathologies in a familiar organ system) and far transfer (explaining pathologies in an unfamiliar organ system). It may be clear that different forms of transfer tend to be defined pragmatically, depending on the context in which they are used. In addition, near and far transfer are most probably points on a continuum, rather than a dichotomy (Barnett & Ceci, 2002).

In search of more domain-independent criteria for near and far transfer to be used in our study, we veered back to Thorndike and Woodworth’s (1901) theory of identical elements. According to Thorndike and Woodworth the more elements, or features, two situations have in common the nearer the transfer from one situation to the other. This similarity principle was recently revived by Gick and Holyoak (2014). We have selected X-rays for our transfer study such, that the test slides either had all elements available that were also available in the training slides, albeit in a different configuration (near transfer), or that not all elements were available in the test slides (far transfer). In the Method section we will further operationalize both forms of transfer tasks.

A key consideration beyond that of transfer is knowledge retention. If someone truly understands the underlying features of the chest x-rays for which they are trained, do they retain this information? The Ebbinghaus forgetting curve, which hypothesises a reduction in memory retention over time (Ebbinghaus, 1885), is widely accepted and the decay in knowledge he observed has been replicated in a number of studies (Murre & Dros, 2015; Schmidt et al., 2000). In their article, Semb and Ellis (1994) explore factors which impact long-term knowledge retention, and one important consideration they discuss is that multiple and distributed opportunities for learning a task are required in order for an improvement in long-term knowledge retention to be observed (Semb & Ellis, 1994). Another key factor is the concept that retrieval of learned information through testing leads to better retention (a phenomenon known as the testing effect), as shown by Roediger and Karpicker in their experiments exploring long-term retention (Karpicke & Roediger, 2007; Roediger & Karpicke, 2006). With this in mind, the current study hoped to show that the training participants receive (which includes repeated exposure to cases as well as the retrieval of diagnoses in test format) promotes a degree of long-term knowledge retention after a two-week hiatus following the initial training phase (although a reduction in knowledge would be expected).

To study transfer and long-term retention of chest X-ray diagnoses, novice medical students in the study described in this paper were presented with eight cases, representing eight different diagnoses. After initial familiarisation, participants were trained in half of the diagnoses. At test, participants were asked to diagnose (1) the four cases they were trained for, (2) near transfer versions for each of the four trained cases, (3) far transfer versions for each of the four trained cases, and (4) the untrained cases in different versions. Accuracy of diagnosis and time needed to arrive at that diagnosis were the dependent variables. Two weeks later, a retention test was conducted. We hypothesised that if participants are able to learn the key diagnostic features of a condition represented on a chest x-ray and are trained for these, then knowledge transfer should occur, but we would expect a reduction in accuracy and time taken to diagnosis as the cases deviate further from the originals. Hence, diagnostic accuracy seen should be highest in trained cases, followed by near then far and finally, lowest in untrained cases, with time taken following a reverse pattern. For the retention test, the same pattern would be predicted although one would expect that some knowledge has been forgotten over time and therefore accuracy scores would be likely to drop.

## Method

### Design

Participants were randomly allocated to the conditions of the experiment in a counterbalanced fashion. Participants received training on 4 of 8 different medical cases. To that end, the cases were subdivided in two groups of four cases, and each participant served as his or her own control condition. The independent variable was thus trained vs. untrained cases and the dependent variables were diagnostic accuracy scores and response time to generate a diagnosis. The ethical approval to conduct the study was granted by the Institutional Review Board (IRB) of Nanyang Technological University, Singapore. Participants were given a $10 coffee voucher for their involvement in the study.

### Participants

A total of 36 s year medical students (67% male, 33% female) with an average age of 20.7 years (SD = 0.89) took part in this study which took place in 2018. The students were from the Lee Kong Chian School of Medicine, Singapore. Of note, one student did not participate in Part 2 of the study, hence N = 35 for Part 2.

### Materials

The materials consisted of a set of 24 online chest x-ray cases (without clinical vignettes), presented to participants using Qualtrics (an online survey tool). The images were obtained from an hospital image bank and were anonymised before use in the study. The diagnoses included were: collapse, consolidation, bulla, emphysema, effusion, lung mass, pneumothorax, and fibrosis. For each diagnosis, there were three images: one original case to demonstrate all the key diagnostic x-ray features of that condition, one which contained the same key features although configured differently (aiming to test for ‘near transfer’), and finally one which missed one or more features and represented other features in different fashion (to allow testing for ‘far transfer’). To define near and far transfer numerically, and in line with prescriptions provided by Thorndike and Woodworth (1901), two authors (LVR and JIR) independently counted the number of deviations from the original slides. Comparing the number of key features among these conditions showed that the original and the near transfer x-rays differed on average with respect to 2.87 features (standard deviation = 1.12), whereas the far transfer x-rays differed on average in 3.88 features (standard deviation = 0.99) from the originals. See “Appendix 1” for all cases, including variations signifying the near- and far-transfer levels.

### Procedure

Part 1 of the study, training and immediate test, took place in a communication skills suite consisting of 21 individual consulting rooms, each with its own desktop computer. Each participant was allocated to their own room. At the start, they were briefly introduced to the study verbally, subsequent instructions were seen on screen. Once participants had completed the online task under strict exam conditions, they were able to leave.

There were three phases to the online exercise: firstly, the familiarisation phase, secondly, the training phase and lastly, the test phase. During the familiarisation phase, participants were exposed to the original eight cases for each condition, which at this stage were annotated to highlight key x-ray features and had accompanying descriptions and a diagnosis attached.

Following the familiarisation phase, the participants were then randomly assigned to either Group 1 or Group 2 for the training phase. Group 1 were to be trained in 4 out of the 8 cases (fibrosis, collapse, bulla, effusion) and Group 2 were to be trained in the other 4 cases (consolidation, pneumothorax, mass, emphysema). This was done to offset possible differences in case difficulty. The training phase cases were the same images used during the familiarisation phase, but now only the image was seen with all descriptions and annotations removed. The images were presented in a random order (each case repeated 10 times) and participants asked to select an answer from a list of 8 diagnoses’ (corresponding to the 8 conditions seen in the familiarisation phase). These answer options were also randomised to ensure there was no recall associated with their position on the screen. In the event that a participant selected an incorrect diagnosis, the familiarisation page for that case would be shown to them again before moving on. The reason for this and the case repetition was to encourage pattern recognition of the features of each condition. After working through the 10 case repetitions of the four cases, participants moved onto the time trial component of the training phase. Participants were exposed to the four same cases again, working through five repetitions of each case. Each repetition had a time limit attached: for the first three repetitions of each case, a diagnosis had to be selected within a 5-s time limit, and for the final two repetitions, the time limit was reduced to 4-s. In the event, an incorrect diagnosis was made or the time limit was exceeded, the familiarisation page for that case was reviewed again before moving on. The time trial element was introduced to ensure that all participants were able to diagnose correctly and instantly all cases for which they were trained.

**Fig. 1:**
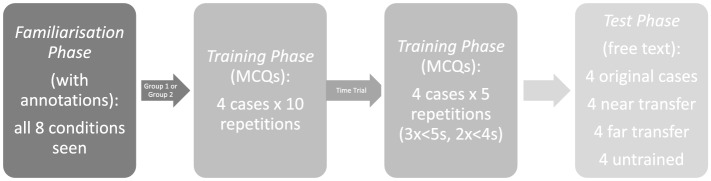
Summary of process, part 1, transfer phase. The test phase below was repeated during part 2, retention phase (methods)

Upon completion of the time-trials, participants moved onto the test phase. During the final test phase participants saw the four trained cases again as well as the corresponding near and far transfer cases for each trained condition (images only) according to their allocated group. They also saw 4 of the transfer cases for the cases they were not trained in, hence these were deemed ‘untrained’. In total, participants saw 16 cases presented in a random order during the test phase (the 4 trained cases, 4 near transfer, 4 far transfer and 4 ‘untrained’). For each case, they were first presented with the image and asked to proceed to the next page as soon they knew the diagnosis. The time taken was recorded. On the next page, they were asked type to their diagnosis in a free text box. These answers were marked by two independent medical doctors. Figure 1 summarises the process of Part 1 visually and “Appendix 2” provides case examples at each phase of the exercise.

Two weeks later, participants were asked to return to the same communication skills suite for Part 2 of the study, the retention test. They were again allocated to individual rooms and completed the exercise under strict exam conditions, which this time was much simpler: a repeat of the test phase from part 1. Their answers were marked as previously and time taken recorded.

### Analysis

Two one-way repeated-measures ANOVAs were conducted for both Part 1 transfer data and Part 2 retention data. For both repeated-measures ANOVA the independent variable was condition (trained versus untrained), and the dependent variable for the first ANOVA was diagnostic accuracy for trained, near-transfer, far-transfer, and untrained cases. For the second ANOVA, the dependent variable was response time.

These analyses enabled us to test whether training for half of the cases resulted in significant enhancements of the participants’ ability to engage in pattern recognition, signified by the increase in diagnostic accuracy and reduced response time for those cases compared to near-transfer, far-transfer and untrained cases. The significance level for all analyses was set to P = 0.05.

## Results

Tables 1 and 2 summarise the descriptive statistics for Part 1 (Transfer Test Phase) and Part 2 (Retention Phase), respectively. The first one-way repeated-measures ANOVA was conducted to explore whether there were significant differences in diagnostic accuracy between the trained, near-transfer, far-transfer and untrained cases.

**Table 1:** Descriptive statistics diagnostic accuracy scores and response time: trained, near-transfer, far-transfer and untrained cases for Part 1, transfer test phase (*M* mean, *SD* standard deviation)

Dependent variable	Trained cases	Near Transfer cases	Far Transfer cases	Untrained cases
Diagnostic accuracy (out of 4) (N = 36)	M = 3.75 SD = 0.44	M = 2.08 SD = 1.18	M = 1.31 SD = 1.14	M = 0.72 SD = 0.85
Time spent on generating diagnosis per case (seconds) (N = 36)	M = 4.95 SD = 3.14	M = 15.77 SD = 1.61	M = 18.80 SD = 13.73	M = 19.46 SD = 13.75

**Table 2:** Descriptive statistics diagnostic accuracy scores and response time: trained, near-transfer, far-transfer and untrained cases for part 2, retention phase (*M* mean, *SD* standard deviation)

Dependent variable	Trained cases	Near transfer cases	Far transfer cases	Untrained cases
Diagnostic accuracy (out of 4) (N = 35)	M = 2.29 SD = 1.02	M = 1.57 SD = 1.15	M = 1.17 SD = 0.95	M = 0.89 SD = 0.80
Time spent on generating diagnosis per case (seconds) (N = 35)	M = 5.74 SD = 2.52	M = 9.52 SD = 5.64	M = 11.03 SD = 8.13	M = 11.86 SD = 8.19

The results revealed that there was a significant main effect: *F*(3, 105) = 152.32, Wilks’ Lambda = 0.07, *P* < 0.001, *η*^*2*^ = 0.93. Planned pair-wise comparisons showed that the diagnostic accuracy score for the trained cases (M = 3.75/4) was significantly higher as compared with all other conditions (*p* < 0.001). The difference in diagnostic accuracy scores between the near- (M = 2.08/4) and far-transfer (M = 1.31/4) conditions was also significant (*p* < 0.001). Finally, the difference in diagnostic accuracy scores between the far-transfer and untrained (M = 0.72/4) conditions was statistically significant as well (*p* = 0.03).

**Fig. 2:**
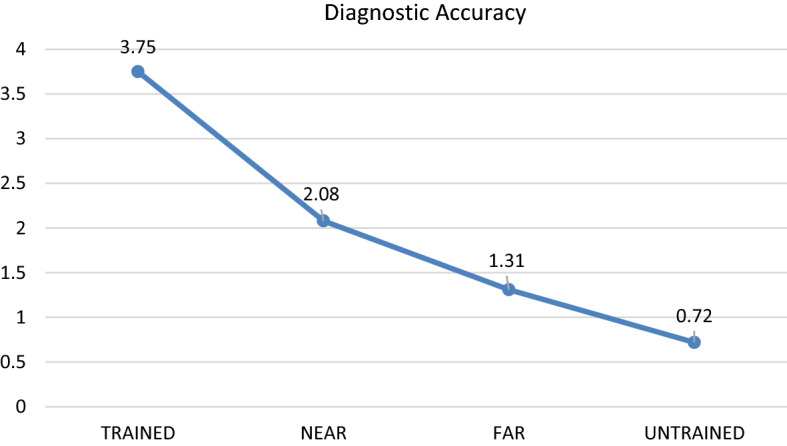
Differences in diagnostic accuracy between trained, near transfer, far transfer and untrained cases for part 1, transfer test phase (mean score out of 4)

**Fig. 3:**
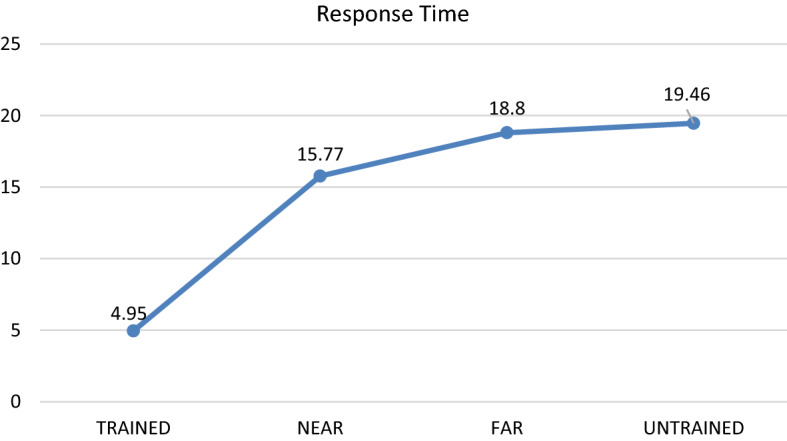
Differences in mean response time per case (in seconds) for diagnosing trained, near transfer, far transfer and untrained cases for part 1, transfer test phase

The second one-way repeated-measures ANOVA was conducted to assess whether there are significant differences in response time for trained, near-transfer, far-transfer and untrained cases. The results revealed that there was a significant main effect: *F*(3, 105) = 17.72, Wilks’ Lambda = 0.38, *P* < 0.001, *η*^*2*^ = 0.62. The results of the planned pair-wise comparisons suggest that the response time for the trained cases (M = 4.95 s per case) was significantly faster than for all other conditions (*p* < 0.001). The resulst of the pair-wise comparisons also revealed that there was a marginally significant difference in terms of response time between the near- (M = 15.77 s per case) and far-transfer cases (M = 18.80 s per case, *p* = 0.06). Finally, there was no significant difference between the far-transfer cases and untrained cases (M = 19.46 s per case, *p* = 0.69). See Figs. 2 and 3 for a visual representation of these results.

The results of the first one-way repeated-measures ANOVA for Part 2 (retention), revealed a similar pattern as with Part 1 (transfer): there was a significant main effect for diagnostic accuracy: *F*(3, 102) = 14.15, Wilks’ Lambda = 0.43, *P* < 0.001, *η*^*2*^ = 0.57. Results of the planned pair-wise comarisons demonstate that the diagnostic accuracy score for the trained cases (M = 2.29/4) was significantly higher as compared with all other conditions (*p* < 0.01). The results also suggest that the difference in diagnostic accuracy scores between the near- (M = 1.57/4) and far-transfer (M = 1.17/4) conditions was marginally significant (*p* = 0.055). Finally, the difference in diagnostic accuracy scores between the far-transfer and untrained (M = 0.89/4) conditions did not reach statistical significance (*p* = 0.20).

**Fig. 4:**
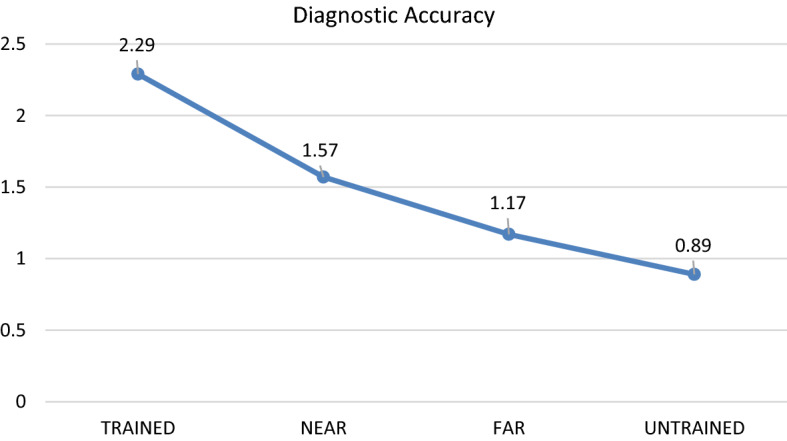
Differences in diagnostic accuracy between trained, near transfer, far transfer and untrained cases for part 2, retention phase (mean score out of 4)

**Fig. 5:**
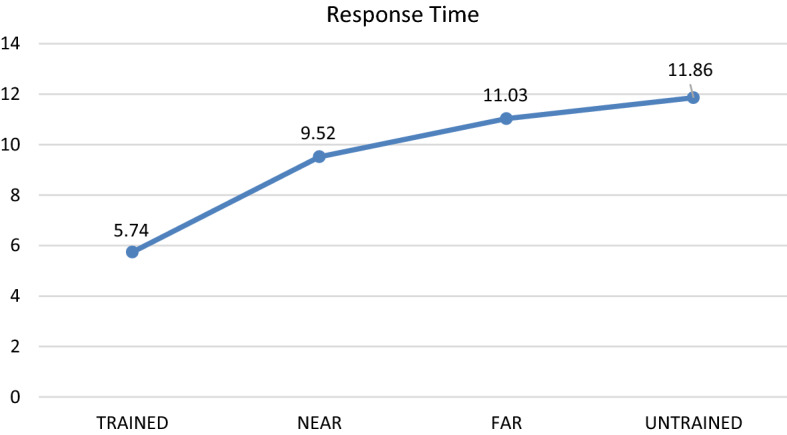
Differences in mean response time per case (in seconds) for diagnosing trained, near transfer, far transfer and untrained cases for part 2, retention phase

With regard to response time, the pattern observed for Part 1 was also retained: There was a significant main effect: *F*(3, 105) = 8.99, Wilks’ Lambda = 0.54, *P* < 0.001, *η*^*2*^ = 0.46. The results of the planned pair-wise comparisons suggest that the response time for the trained cases (M = 2.52 s per case) was significantly faster than for all other conditions (*p* < 0.001). Furthermore, the results suggest that there was no significant difference in terms of response time between the near- (M = 9.52 s per case) and far-transfer cases (M = 11.03 s per case, *p* = 0.16), although the near-transfer cases were diagnosed slightly faster than the far-transfer cases. Finally, there was also no significant difference between the far-transfer cases and untrained cases (M = 11.86 s, *p* = 0.52). See Figs. 4 and 5 for a visual representation of these results.

## Discussion

This study explores the application and retention of knowledge in the context of clinical decision making and dual process theory. In order for successful clinical reasoning to occur, and for a doctor or medical student to come to a correct diagnosis, it is necessary for them to be able to apply the knowledge they have gained in various contexts to other scenarios. In other words, they require the ability to *transfer* knowledge from one situation to another. However, despite its importance in medicine and many other domains, the transfer of knowledge learned is recognized as a process which happens infrequently (Gick & Holyoak, 1980; Haskell, 2001).

This paper builds on a prior study by Rosby et al. (2018), which demonstrated that it was possible to train novice medical students to exhibit expert-like decision-making behaviours, mirroring that of System-1 thinking. However, what this work did not explore was whether the training also promoted the *transfer* and long-term *retention* of this knowledge.

The key objective of the current study was to establish whether students trained for System-1-type performance simply learn to recognize the cases for which they are trained (in which case, transfer of knowledge would *not* occur), *or* are able to learn the underlying diagnostic features of particular chest diseases (as represented on X-ray). The latter would subsequently enable them to apply this knowledge to new representations of the same condition, varying in terms of the features they have in common (Thorndike & Woodworth, 1901). It was expected that as cases diverged from the original, the accuracy in diagnosis would reduce and time taken to reach that diagnosis would increase.

If trained cases had been understood well enough that students could apply and transfer this knowledge, we also predicted that a degree of knowledge retention would occur although recognize that accuracy would likely reduce secondary to knowledge decay as previously discussed in the literature (Ebbinghaus, 1885; Murre & Dros, 2015; Schmidt et al., 2000).

To test these hypostheses, a study was designed and conducted in two parts. During Part 1, in order to test for knowledge transfer, novice medical students’ were presented with eight online chest X-ray cases similar to those described by Rosby et al. (2018). They were shown half of them repetitively, asked to select the correct diagnosis from a list and were provided with remedial training in the event that they were incorrect. Finally, participants were asked to provide free text diagnoses for a total of 16 cases: the four trained cases, four cases to test for near transfer of knowledge, four cases to assess far transfer, and four unrelated, untrained cases, of different diagnoses to those trained for. Part 2 was conducted two weeks later to measure whether there was a degree of knowledge retention as a result of the training received, which include a knowledge retrieval test. Participants were asked to provide diagnoses for the same 16 cases they had seen in the final stage of Part 1. Dependent variables for both parts of the experiment were diagnostic accuracy and processing time.

Overall, the training particpants received did promote knowledge transfer. Participants were able to apply knowledge learned about the trained cases to diagnose new cases of the same diagnosis, differing in similarity of features. This indicates that near transfer and far transfer did indeed occur to varying degrees, although diagnostic accuracy was reduced compared to that of trained cases, coupled with a significant increase in response time, as predicted. Near transfer cases were more accurately and more quickly diagnosed than far transfer cases. Performance in the untrained cases was lower than that of the far transfer cases, although time taken to reach a diagnosis was similar. This pattern shows that the further away from the original case the images are, the more difficult it is to transfer the knowledge learned. There was a degree of successful knowledge retention demonstrated, although diagnostic accuracy scores were reduced as one would expect.

So, what are the possible reasons why we see successful transfer in this study? It is recognised that successful transfer depends upon a wide variety of factors, some of which are fulfilled in this study and will be further discussed. Firstly, transfer is dependent both on context and adequacy in learning (Bransford et al., 1999), as well as the ability of the learner to generalise what they have studied beyond the initial learning event without any new learning taking place (Lobato, 2003). In this case, the context of the study was relevant to the participants, and the exercise provided many opportunities to reinforce the new knowledge being acquired allowing an adequate learning experience. On the other hand, the students saw always the same versions of the particular diseases during the training phase. Perhaps transfer would be enhanced when students, already in the training phase would be confronted with different versions of the same diseases. A future experiment using our paradigm could compare transfer levels as a result of seeing the same cases all the time, versus seeing different cases during training.

Secondly, it is also important to take into account whether there is any correlation between the learning task and the transfer test, in other words whether there is ‘transfer appropriate learning’ occurring (Blaxton, 1989). It is clear in this study that the initial learning task, that of learning diagnostic features of particular chest conditions on X-ray and reinforcing this with additional training, is closely correlated with the test phase, when participants were asked to provide free-text diagnoses’ for a number of chest X-rays, some familiar and others less so.

Furthermore, transfer of knowledge is positively influenced by the motivation of the learner because this affects the quality of the initial phase of learning (Pugh & Bergin, 2006). It is likewise helpful if the learner can appreciate the worth in what they are learning (Anderson et al., 1996). Simply put, if the learner can see beyond the learning event and how it can be used later. This is also the case when it is clear that the information being learned will have an impact on others (Pintrich & Schunk, 2002). All of these factors apply to our participant group and it is fair to assume that they, as medical students, were likely to have been motivated to learn during this exercise given the relevance to their studies and later medical practice with patients.

Let’s now consider the implications of this study. Firstly, to our knowledge, this is one of the a very small number of studies within the field of diagnostic reasoning (Norman et al., 2007) to demonstrate successful knowledge transfer as a result of a training exercise, and is one of the few studies to provide evidence of knowledge transfer in general, even beyond the context studied here. This possibly adds to the body of literature in this field, bringing a fresh perspective and a new paradigm for further investigation.

Another contribution is the demonstration of a degree of long-term knowledge retention resulting from completion of the online training exercise which involved repeated exposure to specific cases and retrieval of diagnoses. This undercuts existing literature which largely suggests that only if a learner undertakes multiple and distributed opportunities for learning a particular task, will sufficient knowledge retention emerge (Semb & Ellis, 1994). It does however provide additional evidence to support results of experiments carried out by Karpicke and Roediger, who showed that repeated studying and prior testing enhanced long-term knowledge retention (Karpicke & Roediger, 2007; Roediger & Karpicke, 2006). In our study, it took less than 8 min on average to train students in chest x-ray interpretation in a one off training exercise, to a degree that was still detectable two weeks later.

These results, in relation to both transfer and retention, indicate that the exercise described could represent a useful way of teaching medical students how to develop clinical reasoning skills, a component which despite its importance is often lacking in medical school curricula due to untertainty about how to address it (Eva, 2005). It is suggested that medical students, despite all the efforts made to prepare them for professional practice, do not receive sufficient exposure to the large variation in which disease presents itself, and therefore may lack essential diagnostic competencies when entering the health care system (Schmidt & Mamede, 2015). Intense and continuous training in the diagnosis of diseases in all their variations may be an appropriate response to this problem.

Special consideration should be given to the fact that we have framed our findings in terms of developing System-1 thinking in our second-year students. In the Introduction section we talked about the possibility of training novice medical students to exhibit expert-like decision-making behaviours. In terms of what the existing literature sees as indicators of System-1 thinking: short decision times and high accuracy (Norman et al., 2014), we seem to have succeeded. System 1 is envisioned as an immediate and effortless response of the mind to situations which it recognizes. Our experimental paradigm, using pictures that could be judged in the blink of an eye, was optimized for such response. However, even in the trained condition and on immediate test, students took on average almost five seconds to arrive at a decision. It seems that even under such circumstances, students engage in some analytical thinking, perhaps as an extra check on the accuracy of their initial diagnosis. On the other hand, a study using the same paradigm and in which oxygenation of the prefrontal cortex was measured using functional near-infrared spectroscopy (a sign that analytical reasoning is involved), suggested that when trained cases were judged, the prefrontal contex was *not* involved (Rotgans et al., 2019).

There are of course limitations to our study, the most obvious being that it is focused on a limited field of medicine, chest radiology, so we cannot simply assume that this will be mirrored in other areas of medical practice. What if we were to use vignets of disease rather than pictures that can be judged in the blink of an eye? Would we find similar effects?

Another is the limited number of cases used—students were trained for only 4 cases, and demonstrated success in knowledge transfer and retention. However, in Hatala et al’s (2003) study which used ECGs, and a greater number of case examples, transfer did not occur as hoped (Hatala et al., 2003). It is therefore worth questioning what might happen to transfer and retention if the number of cases in our study were increased?

A further limitation to bear in mind is that the chest x-rays were considered in isolation. In professional practice, x-rays are a part of the diagnostic process, and the diagnosis seen on the X-ray is made in the context of a patient history, examination and other investigation findings. If this information was available, would this make the task of interpreting the x-ray easier, or perhaps more difficult, and how would this impact the likelihood of knowledge transfer and retention?

So, where do we go from here? We have managed to show that the experiment described promotes transfer of the learned knowledge to similar and less similar cases, and not simply recognition of the images trained for. Next, it would be pertinent to consider using this paradigm with a greater array of cases, perhaps in the context of more patient information, such as history and examination to assess how this affects the participants ability to retain and transfer their new found knowledge. We have also noted that this study focusses on a limited area of medicine—so, could this experiment be extended to other specialities, such as histopathology and dermatology to test how far this training is successful outside of the field of radiology? Additionally, having focused on novice medical students, it would seem appropriate to explore this paradigm in more experienced physicians to assess how far the underlying level of experience affects knowledge transfer.
